# Downregulation of AGR2, p21, and cyclin D and alterations in p53 function were associated with tumor progression and chemotherapy resistance in epithelial ovarian carcinoma

**DOI:** 10.1002/cam4.1530

**Published:** 2018-05-29

**Authors:** Mariana Rezende Alves, Natalia Cruz e Melo, Mateus Camargo Barros‐Filho, Nayra Soares do Amaral, Felipe Ilelis de Barros Silva, Glauco Baiocchi Neto, Fernando Augusto Soares, Louise de Brot Andrade, Rafael Malagoli Rocha

**Affiliations:** ^1^ International Research Center AC Camargo Cancer Center São Paulo Brazil; ^2^ Molecular Gynecology Laboratory Gynecologic Department Federal University of São Paulo São Paulo Brazil; ^3^ Department of Gynecology Oncology AC Camargo Cancer Center São Paulo Brazil; ^4^ Department of Anatomic Pathology A.C. Camargo Cancer Center São Paulo Brazil

**Keywords:** AGR2, cell cycle, epithelial ovarian cancer, IHC, prognosis

## Abstract

Anterior gradient 2 protein belongs to a family of chaperone‐like proteins, namely protein disulfide isomerase. Generally, AGR2 is highly expressed in mucus‐secreting cells and endocrine organs, and in this study, we aimed to evaluate AGR2 and cell cycle molecules in epithelial ovarian cancer and its implications on prognosis. One hundred seventy‐five patient's samples that were diagnosed with primary epithelial ovarian carcinoma were selected. All the patients were treated with platinum‐taxane standard chemotherapy after surgery and CA125 serum levels were routinely determined. Four‐micrometer‐thick sections were processed by immunohistochemistry using an automated immunostainer, Ventana BenchMark AutoStainer with AGR2, cyclin D1, p21WAF1, and p53. Forty‐nine of 167 cases (29.3%) showed strong to moderate cytoplasmic marking of AGR2, and 118 (70.7%) had weak to negative expression. The absence of the AGR2 protein was observed in high‐grade serous carcinoma (*P* < .001) and significantly associated with disease‐free survival (DFS;* P* = .034). The expression of G1‐S phase‐regulatory proteins showed loss of p21 in high‐grade serous carcinoma (*P* < .001) and was related with poor DFS (*P* = .003). Strong and diffuse immunoexpression of p53 plus complete absence of p53 staining was interpreted as likely indicating a *TP53* gene mutation. This result showed worse DFS alone (*P* = .012) and combined with low levels of AGR2 (*P* = .005). The expression profile of AGR2 and cell cycle proteins here presented was showed as good prognosis marker in epithelial ovarian cancer. This finding suggests AGR2 and as putative biomarker of disease progression in chemotherapy‐treated high‐grade serous carcinoma patients.

## 1 INTRODUCTION

Ovarian cancer is one of the deadliest gynecological malignancies worldwide with 22 280 new cases and 14 240 estimated deaths in the United States.^1^ Historically, treatment has involved surgery combined with platinum‐based chemotherapy.^2^ The main factors affecting patient's prognosis are advanced stage at diagnosis and primary or secondary chemotherapy drug resistance, especially for that persistent or recurrent ovarian carcinoma.^3^ The *TP53* gene, encoding the p53 tumor suppressor, is the most frequent target for mutation in human cancer.^4^ Of the currently known molecular alterations in ovarian carcinoma, loss of p53 function is the most common one,^5^, ^6^ and some studies describe AGR2 (anterior gradient 2 protein) inhibits p53 activity.^7^, ^8^

AGR2 is a small 154 amino acid protein with a single central Cys residue, which belongs to a family of chaperone‐like proteins, namely protein disulfide isomerase (PDI).^9^ PDI‐related proteins are microenvironmentally regulated proteins that can catalyze the formation, reduction, or isomerization of disulfide bonds in their network. These enzymatic reactions facilitate protein maturation into bioactive conformation in the endoplasmic reticulum (ER).^10^ Generally, AGR2 is highly expressed in mucus‐secreting cells and endocrine organs, such as lung, stomach, colon, prostate, and small intestine,^11^, ^12^, ^13^ from an evolutionary perspective, believed to be involved in the epithelial barrier function^11^, ^14^ especially on AGR2 losses, which elevates ER stress in some of the intestine cell types, providing the first evidence for AGR2 function in ER‐dependent protein folding.^14^

In tumorigenesis, AGR2 plays an important role by activating survival and metastasis pathways by interacting with cyclin D1, cathepsin B, D, Myc, p‐Src, and EGFR and by blocking cell death inhibiting p53 function. The overexpression of AGR2 was observed in several tumor types,^15^, ^16^, ^17^ which supports the hypothesis of AGR2 be an oncogene, but the downregulation of this gene also was observed in prostate tumors,^18^ ovarian,^19^ colorectal,^20^ and pancreas.^21^

Despite the existing knowledge that AGR2 may interact with p53,^7^, ^22^ little is known about its possible relation with cell cycles proteins and prognostic value in ovarian cancer context. In order to evaluate the association of AGR2 and cell cycle molecules in ovarian cancer and its implications on prognosis, we have used immunohistochemistry to investigate protein expression of AGR2, p21, cyclin D, and p53. Our study showed that the downregulation profile of AGR2, p21, and cyclin D, and alterations of p53 function were associated with tumor aggressiveness characteristics such as progression, invasion, relapse, and worse disease‐free survival.

## 2 MATERIALS AND METHODS

### 2.1 Clinical samples

One hundred seventy‐five samples from patients diagnosed with primary epithelial ovarian carcinoma between 2003 and 2013 were selected from the Anatomic Pathology Department of AC Camargo Cancer Center (São Paulo, Brazil). All patients provided written informed consent for the collection of samples and subsequent analysis, and this study was approved by the Ethics Committee of the institution. Patients were treated with platinum‐taxane standard chemotherapy after surgery and CA125 serum levels were routinely determined to evaluate response. Staging of the disease was assessed according to FIGO criteria, optimal debulking surgery was defined as less than 1 cm of gross residual disease, and suboptimal debulking surgery was defined as more than 1 cm of residual disease.

Recurrence was defined per GCIG criteria after evaluating RECIST and CA125 progression in the medical charts, and the date of the earlier event was considered for progression. CA125 progression by GCIG is considered if there is a doubling in CA125 from the upper limit of normal and for those patients whose CA125 never fell to the normal range, a doubling from the nadir is considered progression.^23^

Disease‐free survival (DFS) was calculated as the interval from primary surgery to disease progression or recurrence. Platinum‐free interval (PFI) was considered the interval between the date of the last platinum compound infusion and the date of disease progression. Platinum‐resistant recurrence was defined as recurrence with a PFI of<6 months. Overall survival was determined by the time interval between the date of diagnosis and the date of death due to ovarian cancer.

The clinical data obtained from the medical records included age at diagnosis, type of surgery, volume of residual disease after surgery (1 cm or >1 cm), tumor stage, date of last platinum‐based chemotherapy, and date of recurrence after each treatment until last follow‐up. The histologic characteristics of surgically resected specimens were reviewed by expert gynecopathologist (LBA) in order to confirm the histologic subtype. Pathological data, such as impairment of ovarian surface and fallopian tube, bilateral involvement, lymphatic and blood vascular invasion, and lymph node involvement, were evaluated. Histologic subtype was diagnosed based on most recent WHO classification of ovarian tumors. Formalin‐fixed paraffin‐embedded samples were used for the construction of a tissue microarray.

### 2.2 IHC analysis

Representative tumor areas were marked on HE‐stained slides by the pathologist, and two tissue cores (diameter 1 mm) subsequently punched out the respective donor paraffin blocks using a tissue microarray. Four‐micrometer‐thick sections were processed by immunohistochemistry using an automated immunostainer (Ventana BenchMark AutoStainer) with polyclonal anti‐human AGR2 antibody (1:100 ABGENT) and monoclonal antibody: Cyclin D1 rabbit (clone EP12; ready to use A. MENARINI), p21WAF1 (clone DCS‐60.2; ready to use A. MENARINI), and p53 mouse anti‐human antibody DO‐7 (dilution 1:100; Dako), suitable for detecting both wild‐type and mutant p53 protein, were used as the primary antibody. The antibodies were incubated at 37°C for 32 minutes. Specific staining was detected by applying an enzyme (with a corresponding substrate‐chromogen system—HRP) directly conjugated to the secondary antibody. The complex was then visualized with hydrogen peroxide substrate and 3, 3′‐diaminobenzidine tetrahydrochloride (DAB) chromogen, counterstained with hematoxylin, and mounted for microscopic examination. All batches included positive controls, and omission of primary antibody was used as negative control.

Scores are expressed as a percentage of positive nuclear staining within representative areas of the tumor sample. Cyclin D1 and p21 expression, analyzed in 162 and 158 respectively according to tissue availability of interpretation, were scored by percentage of positive cells, as follows: 0 (absence positive cells or until 10% of positive cells), 1 (11%‐49% of positive cells, irregular stain), and 2 (≥50% of positive cells, diffuse stain).^24^ The categories 0 was categorized as low level and 2 and 3 were considered as high level of staining. p53 expression had 166 conclusive samples able to perform the analysis, which positive staining was defined as a homogeneous pattern of strong and diffuse nuclear staining. The absence of staining was considered negative, and patchy patterns of nuclear staining interspersed with negative staining were considered p53 on basal levels.^25^ To AGR2 evaluation it was performed in 167 tissue samples, and the percentage of positive cytoplasmic tumor cells it was scored as established by the percentage of positive cells (PPC) and intensity of immunostaining (II) [HSCORE = (PPC × II), varied from 0% to 100%, and intensity of immunostaining (II), varied from 0 (negative staining), 1 (weak staining), 2 (moderate staining), and 3 (strong staining), in which [HSCORE = (PPC × II), with a ranking between 0 and 300.^26^ Low levels of AGR2 were categorized as 0 to 100 and high levels as 101 to 300.

### 2.3 Public available databases

To integrate the data obtained by IHC, we analyzed the expression of AGR2, p53, p21, and cyclin D1 in public available databases: (1) proteome data from RPPA (reverse phase protein array) for *TP53* (p53), *CDKN1A* (p21), *and CCND1* (cyclin D1), obtained from TCGA ovarian carcinoma patients, downloaded from cBioPortal (http://www.cbioportal.org/); (2) mass spectrometry data from Ovarian Serous Cystadenocarcinoma cancer downloaded from Clinical Proteomic Tumor Analysis Consortium (CPTAC; https://cptac-data-portal.georgetown.edu/cptacPublic/), available only for *AGR2 and TP53;* and 3—exome sequencing data of *TP53* (somatic gene‐level nonsilent mutation) from TCGA ovarian carcinoma patients, downloaded from cBioPortal. All the remained details are available on supplementary data (Appendix S1).

### 2.4 Statistical analysis

The database was generated in SPSS, version 21.0. The association between categorical variables was analyzed by Chi‐square or Fischer's exact test. Survival curves were determined by Kaplan‐Meier method, and the comparison of survival curves for each variable category was performed using log‐rank test. A univariate analysis was performed by Cox regression. For all tests, *P* ≤ .05 was considered statistically significant.

## 3 RESULTS

### 3.1 Sample characteristics

We evaluated 175 ovarian cancer samples with a median age of 56 years (minimum of 17 years and maximum of 83 years old). According to histologic classification, serous tumors was the most frequent subtype, n = 126 (71.9%). One hundred thirty‐two (77.2%) patients were diagnosed in advanced stages of the disease (III‐IV) and 124 (71%) of the patients had recurrence of the disease, which 44 (28.2%) patients had platinum‐free interval less than 6 months. The remaining clinicopathological features of all 175 patients are described in Table 1.

**Table 1:** Clinicopathologic data of 175 patients with ovarian cancer analyzed in FFPE samples

Variables	Category	N/Total (%)
Histology	HGSOC^a^	114/175 (65.1)
	Others^b^	61/175 (34.9)
Lymphatic vascular invasion	No	59/117 (50.4)
	Yes	58/117 (49.6)
Blood vascular invasion	No	102/117 (87.2)
	Yes	15/117 (12.8)
Bilateral involvement	No	48/115 (41.7)
	Yes	67/115 (58.3)
Necrosis	Absence	69/141 (48.9)
	Presence	72/141 (51.1)
Impairment of ovarian surface	No	24/103 (23.3)
	Yes	79/103 (76.7)
Metastatic lymph node	No	59/109 (54.1)
	Yes	50/109 (45.9)
Surgery	Primary debulking surgery	133/173 (76.9)
	Interval debulking surgery	40/173 (23.1)
Residual disease	<1 cm	107/153 (69.9)
	>1 cm	46/153 (3.0.1)
Staging	I/II	39/171 (22.8)
	III/IV	132/171 (77.2)
ECOG	0	69/139 (49.6)
	1.2.3	70/139 (5.0.4)
Chemotherapy	Neoadjuvant	35/152 (23)
	Adjuvant	117/152 (77)
Death	No	88/175 (50.3)
	Yes	87/175 (49.7)
Relapse	No	49/173 (28.3)
	Yes	124/173 (71.3)

a HGSOC: high‐grade serous ovarian carcinoma.

b Others: serous borderline, low‐grade serous carcinoma staining, clear cell, endometrioid, mucinous, mixed epithelial tumor, and carcinosarcoma.

### 3.2 AGR2 expression is associated with less aggressive tumors and best prognosis

**Figure 1:**
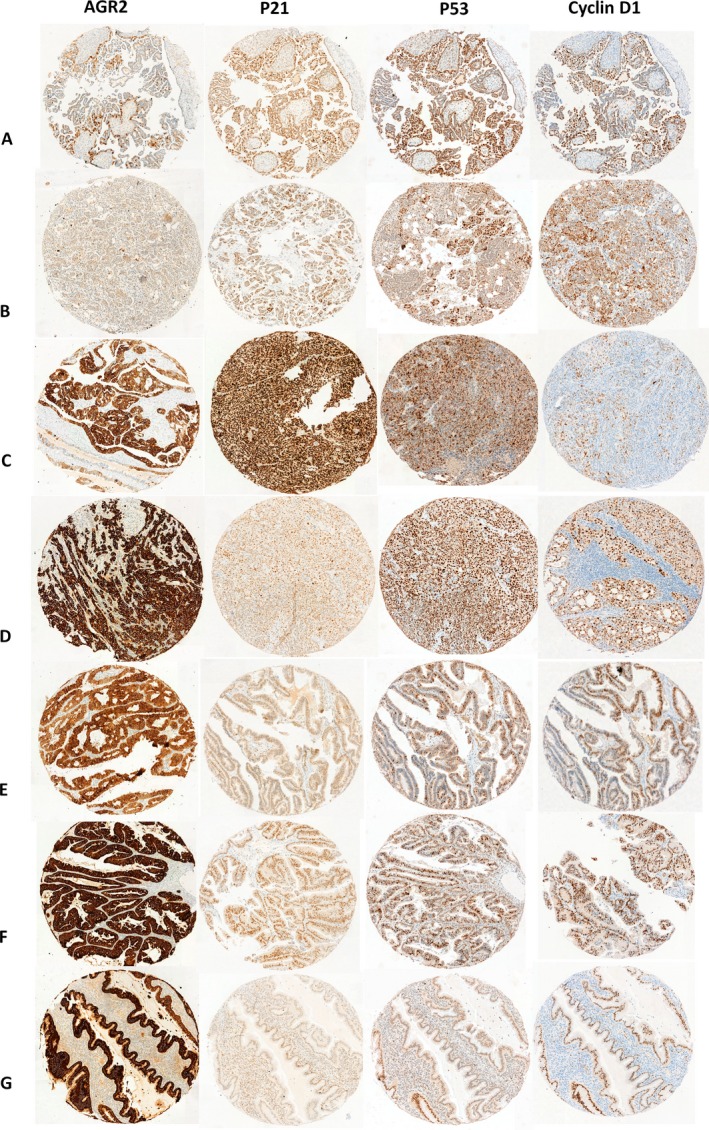
Marker expression by immunohistochemistry of AGR2, p53, p21, and cyclin D1 markers presenting on ovarian cancer among subtypes: A, serous borderline; B, low‐grade serous carcinoma staining; C, high‐grade serous carcinoma; D, clear cell; E, endometrioid; F, mucinous, and G, carcinosarcoma. All the images at 5× magnification

One hundred sixty‐seven samples were analyzed for this marker that showed a strictly cytoplasmic staining (Figure 1). Forty‐nine of 167 cases (29.3%) showed strong to moderate cytoplasmic marking of AGR2 and 118 (70.7%) had weak to negative expression. The absence of the AGR2 protein was observed differentially expressed in high‐grade serous carcinoma (<0.001), samples with lymphatic vascular invasion (*P* = .003), bilateral involvement (*P* = 0.005) necrosis (*P* = .034), ovarian surface involvement (*P* = .032), and lymph node metastasis (*P* = .003). Regarding the response to treatment, the absence of the protein corresponded to 75.9% of patients that relapsed (*P* = .049; Table 2).

**Table 2:** Distribution of ovarian cancer samples according to the presence or absence of AGR2, p53, P21, and cyclin D1 protein and standard prognostic factors

Variables	Category	AGR2			p53			p21			Cyclin D1		
		n (%)		*P*‐value	n (%)		*P*‐value	n (%)		*P*‐value	n (%)		*P*‐value
		Absence	Presence		Absence	Presence		Absence	Presence		Absence	Presence	
Histology	HGSOC	95 (80.5)	14 (28.6)	**<.001** b	12 (26.7)	101 (83.5)	**<.001** b	95 (77.2)	8 (22.9)	**<.001** b	101 (74.8)	9 (33.3)	**<.001** b
	Others^a^	23 (19.5)	35 (71.4)		33 (73.3)	20 (16.5)		28 (22.8)	27 (77.1)		35 (25.2)	18 (66.7)	
Lymphatic vascular invasion	No	28 (38.9)	28 (70)	**.003** b	22 (71)	32 (40.5)	**.008** b	31 (38.8)	22 (73.3)	**.003** b	39 (44.3)	15 (75)	**.026** b
	Yes	44 (61.1)	12 (30)		9 (29)	47 (59.5)		49 (61.3)	8 (26.47)		49 (55.7)	5 (25)	
Blood vascular invasion	No	58 (80.6)	39 (97.5)	**.026** b	30 (96.8)	65 (82.3)	.063	69 (86.3)	27 (90)	.754	75 (85.2)	20 (100)	.146
	Yes	14 (19.4)	1 (2.5)		1 (3.2)	14 (17.7)		11 (13.8)	3 (10)		13 (14.8)	0 (0)	
Bilateral involvement	No	22 (31.9)	25 (61)	**.005** b	22 (73.3)	24 (30.8)	**<.001** b	24 (31.2)	21 (67.7)	**.001** b	29 (34.1)	16 (76.2)	**.001** b
	Yes	47 (68.1)	16 (39)		8 (26.7)	54 (69.2)		53 (68.8)	10 (32.3)		56 (65.9)	5 (23.8)	
Necrosis	Absence	36 (40.9)	29 (61.7)	**.034** b	27 (73)	34 (35.8)	**<.001** b	36 (37.5)	22 (71)	**.002** b	44 (41.1)	17 (77.3)	**.004** b
	Presence	52 (59.1)	18 (38.3)		10 (27)	61 (64.2)		60 (52.5)	9 (29)		63 (58.9)	5 (22.7)	
Impairment of ovarian surface	No	11 (16.7)	13 (38.2)	**.032** b	13 (46.4)	10 (13.9)	**.001** b	12 (16)	11 (45.8)	**.006** b	15 (18.8)	7 (41.2)	.058
	Yes	55 (83.3)	21 (61.8)		15 (53.6)	62 (86.1)		63 (84)	13 (54.2)		35 (81.3)	10 (58.8)	
Metastatic lymph node	No	11 (16.7)	13 (38.2)	**.003** b	19 (59.4)	37 (51.4)	.589	35 (46.7)	20 (76.9)	**.015** b	45 (52.9)	10 (62.5)	.667
	Yes	55 (83.3)	21 (61.8)		3 (40.6)	35 (48.6)		40 (53.3)	6 (21.1)		40 (47.1)	6 (37.5)	
Surgery	Primary debulking surgery	84 (72.4)	44 (89.8)	**.025** b	38 (84.4)	87 (73.1)	.188	90 (74.54)	31 (88.6)	.123	100 (75.2)	21 (77.8)	.968
	Interval debulking surgery	32 (27.6)	5 (10.2)		7 (15.7)	32 (26.9)		31 (25.6)	4 (11.4)		33 (24.8)	6 (22.2)	
Residual disease	<1 cm	71 (68.3)	32 (78)	.334	32 (80)	72 (67.9)	.218	69 (65.7)	29 (87.9)	.026	85 (71.4)	15 (65.2)	.728
	>1 cm	33 (31.7)	9 (22)		8 (20)	34 (32.1)		36 (34.3)	4 (12.1)		34 (28.6)	8 (34.8)	
Staging	I/II	17 (14.9)	22 (44.9)	**<.001** b	18 (40.9)	18 (15.3)	**.001** b	18 (15.1)	18 (51.4)	**<.001** b	27 (20.6)	9 (33.3)	.237
	III/IV	97 (85.1)	27 (55.1)		26 (59.1)	100 (84.7)		101 (84.9)	17 (48.6)		104 (79.4)	18 (66.7)	
ECOG	0	42 (45.2)	26 (66.7)	.039	22 (59.5)	45 (46.9)	.268	42 (43.8)	23 (76.7)	.003	55 (50.9)	11 (52.4)	1
	1.2.3	51 (54.8)	13 (33.3)		15 (40.5)	51 (53.1)		54 (56.3)	7 (23.3)		53 (49.1)	10 (47.6)	
Chemotherapy	Neoadjuvant	79 (75.2)	35 (89.7)	.094	28 (77.8)	83 (76.1)	1	86 (78.9)	24 (85.7)	.588	93 (77.5)	16 (72.7)	.832
	Adjuvant	26 (24.8)	4 (10.3)		8 (22.2)	26 (23.9)		23 (21.1)	4 (14.3)		27 (22.5)	6 (27.3)	
Death	No	57 (48.3)	27 (55.1)	.529	29 (64.4)	55 (45.5)	**.045** b	55 (44.7)	26 (74.3)	**.004** b	73 (54.1)	15 (55.6)	1
	Yes	61 (51.7)	22 (44.9)		16 (35.6)	66 (54.5)		68 (55.3)	9 (25.7)		62 (45.9)	12 (44.4)	
Relapse	No	28 (24.1)	20 (40.9)	**.049** b	19 (42.2)	27 (22.7)	**.022** b	25 (20.7)	21 (60)	**<.001** b	31 (23.3)	14 (51.9)	**.006** b
	Yes	88 (75.9)	29 (59.2)		26 (57.8)	92 (77.3)		96 (79.3)	14 (40)		102 (76.7)	13 (48.1)	

HGSOC, high‐grade serous ovarian carcinoma.

a Others: serous borderline, low‐grade serous carcinoma staining, clear cell, endometrioid, mucinous, mixed epithelial tumor and carcinosarcoma.

b Bold values: statistically significance (*P* ≤ 0.05) calculated by Chi‐square or Fischer’s exact test.

Kaplan‐Meier curves showed the importance of AGR2 expression in patients’ outcome. The median of DFS for patients whose tumors presented positivity for AGR2 was 44 months, which was significantly higher than the median survival of patients whose tumors lacked AGR2 expression, which was 22 months (*P* = .034; Figure S1). OS showed no difference between the curves (*P* = .227; Table 3). A Cox proportional hazards regression model showed that the absence of AGR2 protein expression in the tumor was a strong predictor of poor DFS (HR: 0.631; 95% confidence interval: 0.412‐0.966; *P* = .034; Table 4).

**Table 3:** Univariate log‐rank test of protein for overall survival and disease‐free survival rate in 5 years

Features		Overall survival			Disease‐free survival		
		Number of patients	Survival rates (%)	*P*‐value	Number of patients	Survival rates (%)	*P*‐value
AGR2	High	49	32 (65.3)	.227	49	22 (44.9)	**.034** a
	Low	118	32 (65.3)		114	29 (25.4)	
Cyclin D1	High	27	15 (55.6)	.601	27	14 (51.9)	.135
	Low	135	76 (56.3)		131	33 (25.2)	
p21	High	35	26 (74.3)	.072	35	21 (60)	**.003** a
	Low	123	65 (52.8)		119	28 (23.5)	
p53	Basal	45	30 (66.7)	.176	44	21 (47.7)	**.012** a
	Alteration	121	64 (52.9)		118	28 (23.7)	

a Bold values: statistically significance (*P* ≤ 0.05) calculated by log‐rank test.

**Table 4:** Univariate Cox regression analysis for overall survival and disease‐free survival

Feature		Overall survival		Disease‐free survival	
		HR (95% CI)	*P*‐value	HR (95% CI)	*P*‐value
AGR2	High	1	.399	1	**.034** a
	Low	0.810 (0.497‐1.321)		0.631 (0.412‐0.966)	
Cyclin D1	High	1	.712	1	.095
	Low	1.118 (0.617‐2.027)		0.610 (0.342‐1.089)	
p21	High	1	**.035** a	1	**.003** a
	Low	.473 (0.236‐950)		0.423 (0.241‐0.741)	
p53	Basal	1	.088	1	**.029** a
	Alteration	1.609 (0.931‐2.779)		1.626 (1.050‐2.517)	

a Bold values: statistically significance (*P* ≤ 0.05) calculated by Cox regression model. CI, confidence interval; HR, hazard ratio.

### 3.3 Expression of G1‐S phase‐regulatory proteins and the association with clinicopathological parameters

The expression of G1‐S phase‐regulatory proteins was analyzed using cyclin D1, p21WAF1/Cip, and p53 proteins. Cyclin D1 was analyzed in 162 cases and the majority of cases showed a nuclear staining, but interestingly 27 (16.7%) of the samples showed nuclear and cytoplasmic staining. Weak cyclin D1 staining was observed in high‐grade serous carcinoma (*P* < .001), lymphatic vascular invasion (*P* = .026), bilateral involvement (*P* < .001), necrosis (*P* = .004), and patients with recurrence (*P* = .006).

p21 had a specific, sharp, and well‐localized nuclear and cytoplasmic staining pattern, strongly staining in 35 (22.2%) of the 158 cases analyzed. Loss of p21 was significant in high‐grade serous carcinoma (*P* < .001), lymphatic vascular invasion (*P* = .003), bilateral involvement (*P* = .001), necrosis (*P* = .002), ovarian surface involvement (*P* = .006), lymph node metastasis (*P* = .015), stage (*P* < .001), death (0.004), and patients with recurrence (*P* = .006).

p53 expression was evaluated in 166 samples where 67 of 166 (40.4%) presented strong and diffuse marking, 45 of the 166 samples had patchy patterns of nuclear staining, and 54 of 166 (32.5%) presented negative staining. It is already established that the DO‐7 clone p53 surrogates a mutational status in the diagnostic workup of ovarian carcinomas.^27^ Strong and diffuse immunoexpression of p53 plus complete absence of p53 staining is generally interpreted as likely indicating a *TP53* gene mutation.^24^, ^28^ We grouped the results of the protein analysis according to their mutational status and correlate them with clinicopathological features. We observed that p53 is present in 101 of 166 (83.5%) of the high‐grade serous carcinoma samples and 20 of 53 (16.5%) in other histology (*P* < .001). p53 staining based on its mutational status was observed differentially expressed in high‐grade serous carcinoma (*P* < .001), lymphatic vascular invasion (*P* = .008), bilateral involvement (*P* < .001), necrosis (*P* < .001), ovarian surface involvement (*P* = .001), stage (*P* = .001), death (*P* = .045), and recurrence (*P* = .022).

Regarding Kaplan‐Meier analysis, lower levels of cyclin D1 showed a median of DFS of 23 months; however, it did not show difference on survival curves (*P* = .135) and overall survival (*P* = .601). p21 survival curves showed a difference between lower levels of staining and DFS (*P* = .003) and a marginal significance on OS (*P* = .071). Using a classification as aberrant (excessive or completely negative) and basal levels, p53 expression showed a significant prognostic factor for DFS in our group of patients (*P* = .012) but no difference on OS (*P* = .176; Figure S1). Univariate Cox regression analysis showed that the lower levels of p21 protein expression in the tumor were a strong predictor of OS survival (HR: 0.473; 95% confidence interval: 0.236‐0.950; *P* = .035) and DFS (HR: 0.423; 95% confidence interval: 0.241‐0.741; *P* = .003). The remained protein values of Cox proportion hazards model are summarized in Table 4.

### 3.4 Association of AGR2 and G1‐S phase‐regulatory proteins

**Figure 2:**
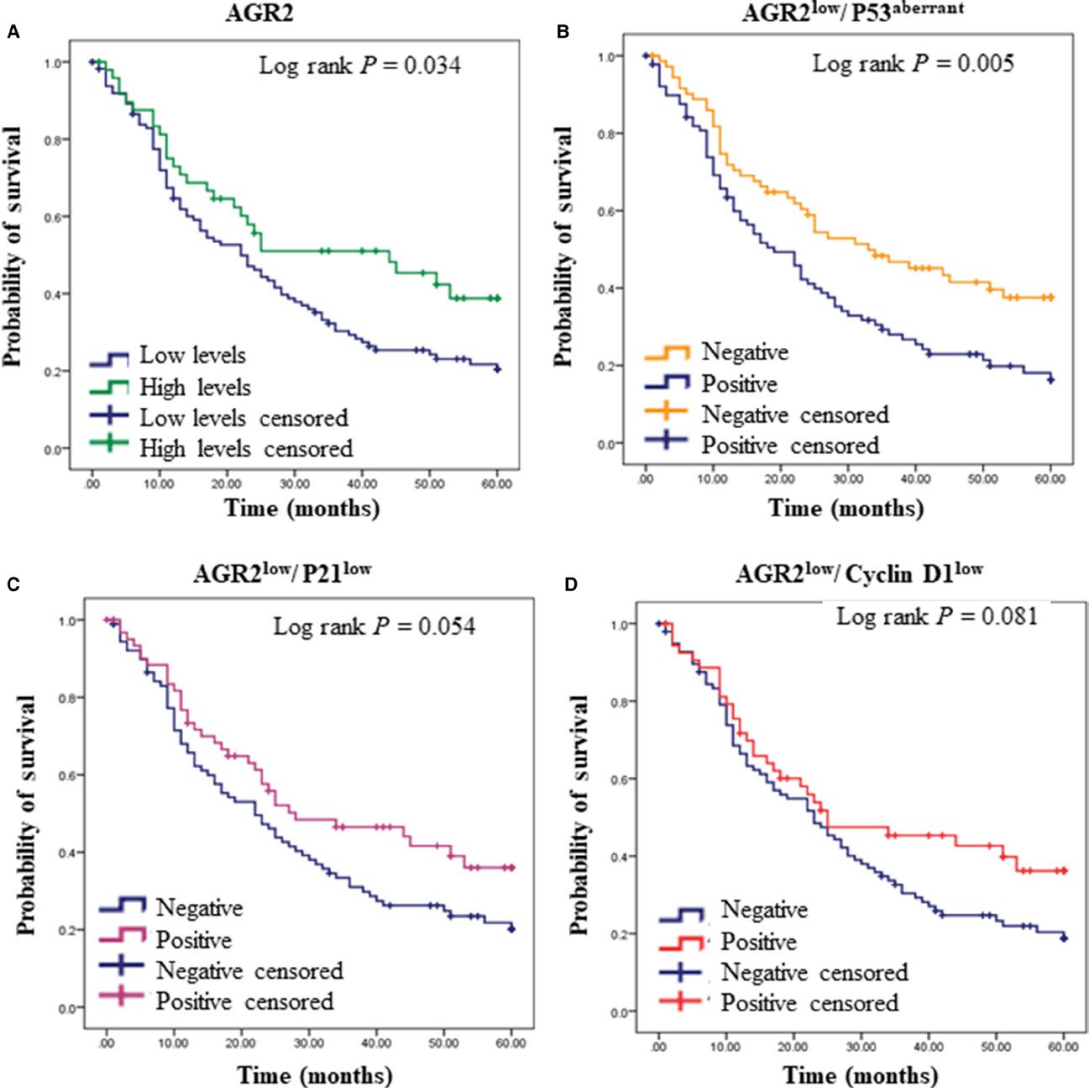
Kaplan‐Meier curve for disease‐free survival for A, AGR2 low and high levels; B, comparison of protein expression levels between AGR2^low^/p53^aberrant^; C, AGR2^low^/p21^low^, and D, AGR2^low^/cyclin D1^low^. All *P*‐values calculated by the log‐rank test

The Chi‐square test for independence was used to discover whether there is an association between AGR2 and the cell cycle proteins. It is shown in Table 5 that the strength of association between AGR2 and cyclin D1, and p21 and p53 is strong. Taking these results together, we tested the pattern of expression of AGR2 low levels with cyclin D1 low levels, p21 low levels, and p53 alterations, respectively, on survival analysis. Each pattern of expression was calculated separately according to DFS (Figure 2) and AGR2^low^/cyclin D1^low^ showed a certain trend toward significance on the survival curves (*P* = .081) DFS analysis; AGR2^low^/p21^low^ presented a marginal trend toward significance (*P* = .054); and AGR2^low^/p53^aberrant^ a strong difference between the curves (*P* = .005) on DFS.

**Table 5:** Association of AGR2 expression and cell cycle proteins

Variables	Category	n (%)		*P*‐value
		Absence	Presence	
Cyclin D1	No	101 (87.1)	31 (72.1)	**.046** a
	Yes	15 (12.9)	12 (27.9)	
p21	No	94 (85.5)	27 (58.7)	**.001** a
	Yes	16 (14.5)	19 (41.3)	
p53	Basal	24 (20.5)	20 (45.5)	**.003** a
	Alteration	93 (79.5)	24 (54.5)	

a Bold values: statistically significance (*P* ≤ 0.05) calculated by Chi‐square or Fischer’s exact test.

### 3.5 Protein analysis of public data

**Figure 3:**
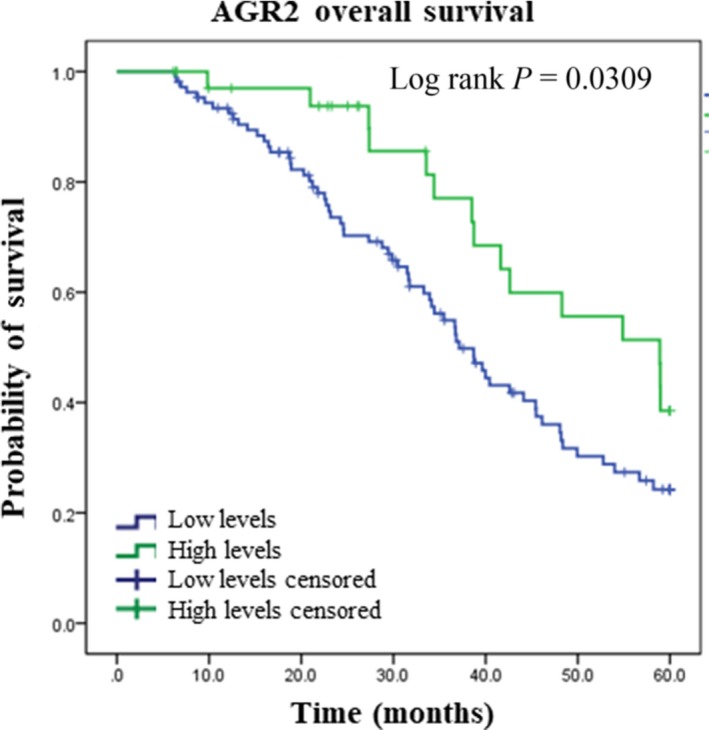
Kaplan‐Meier curve for AGR2 overall survival by mass spectrometry analysis from Clinical Proteomic Tumor Analysis Consortium (CPTAC)

A publically available data set was used to examine AGR2, cyclin D1, p21, and p53 protein expression in human ovarian cancer. The data set was generated using three platforms, and these analyses were not able to confirm our findings. However, AGR2 overall survival, by mass spectrometry analysis, showed a significantly higher survival rates (58.3%) compared with patients whose tumors lacked AGR2 expression (40.2%; *P* = .0309; Figure 3). The additional 213 cases of p53 exome sequencing were performed to support the IHC evaluation. The results showed a predominant p53 mutant in the ovarian cancer cases (n = 188), but its values did not provide any information on survival analysis (Appendix S1).

## 4 DISCUSSION

The increase in AGR2 has been associated with phenotypes such as cell viability, invasion, and metastasis in various human cancers,^15^, ^16^, ^17^ however, in our study, the expression of AGR2 evaluated by IHC was associated with better prognosis, suggesting a versatility in the function of this protein in cancer pathophysiology. Here, we observed that the lower levels of AGR2 protein in ovarian carcinoma were correlated with high‐grade serous carcinoma and characteristics of malignancy, such as lymphatic vascular invasion, bilateral involvement, necrosis, ovarian surface involvement, lymph node metastasis, and relapse (Table 2). We found an association between histology (*P* < .001; Figure 1), which corroborates with Ames and colleagues, who observed a greater expression of AGR2 in endometrioid and mucinous subtypes when compared to the high‐grade serous carcinoma, and absence of AGR2 was associated with increased malignancy, tumor progression, and relapse.^29^

The functional role of increasing AGR2 in promotion oncogenesis has been established by the activation of survival pathways and metastasis,^22^, ^30^ but little is known about how the reduction in AGR2 would act facilitating tumor progression. Some functional studies show the importance of AGR2 in cell differentiation and suggest that low levels of AGR2 promote reduction in protein adhesion expression 21, 31 and increases resistance to apoptosis,^31^ which could justify the aggressive phenotype of our results, which allows the ovarian carcinoma cell to establish an outpost on the omentum and peritoneum.

In an elegant review, Chevet and colleagues described AGR2 biological pathways, which AGR2 is presented in mechanism as a mediator of tamoxifen drug resistance in human breast cancer^32^ in esophageal cancer progression,^7^ and stimulating key cancer‐signaling pathways, such as cyclin D1, metastasis, and others.^22^, ^30^ In esophageal metaplasia tissue, Pohler and colleagues identified a novel p53 inhibitors—AGR2, which was validated as a potent inhibitor of p53‐dependent transcription and a growth‐promoting proto‐oncogene.^7^ In our results, p53 analysis, called as p53 aberration, was observed in 121 samples (72.9%) in which, 101 was classified as high‐grade serous carcinoma, the most frequent subtype in our casuistic (n = 114; Table 2).

Sequencing of DNA tumor is the gold standard to detect different types of p53 mutation and relate these to clinical outcome. Determining the true prevalence of *TP53* mutation is critical for understanding the pathogenesis of ovarian tumor, especially high‐grade serous carcinoma.^5^, ^6^ The rationale behind this interpretation of immunohistochemistry is that inframe point mutations of p53 alter the conformation of the protein and prolong its biological half‐life, thus intensifying the immunohistochemical staining result. Other mechanisms that abolish activity (eg, homozygous deletion of the gene or truncating mutations) may result in loss of p53 protein and are identified as the absence of staining using sensitive immunohistochemical assay.

An independent test showed that the levels of AGR2 differ from the presence of aberrant p53 (Table 5). Survival curves (Figure 2) also could illustrate this hypothesis, showing that low levels of AGR2 together with p53 aberrant show worse DFS (*P* = .005). The ability of AGR2 to suppress p53 activity could be explained, in part, in an environment or tumor which is lower frequency of p53 mutations, for example breast cancer,^33^, ^34^ a disease where AGR2 is overexpressed and p53 wild‐type is maintained in a majority of cases.^8^, ^35^, ^36^ Conversely, where p53 has a high frequency of mutation, as ovarian cancer,^5^, ^6^ the suppression of p53 from AGR2 pathway may be less active.^7^, ^29^

p53 pathway can negatively regulate the cell cycle through the activation of p21.^37^, ^38^ As a proposed investigation of cell cycle molecules, reduction in p21 it was associated with high‐grade serous carcinoma subtype, and anatomopathological characteristics of lymphatic vascular invasion, bilateral involvement, necrosis, ovarian surface involvement, tumor stage, death, and relapse (Table 2 and Figure 2). Inactivation of p53 function leads to loss of p21 induction and impairment of cyclin/CDK complex inhibition. Indeed, we found that downregulation of p21 was significantly associated with poor survival and a shorter disease‐free survival interval, as previously demonstrated by others.^39^, ^40^

Cyclin D1 is also positively regulated by AGR2, and consequently, the reduction in AGR2 induces a decrease in cyclin D1 expression.^29^, ^41^ Cyclin D1 and its binding partners CDK4/6 partially regulate the cell cycle by promoting the G1 to S transition in the cell cycle.^42^ Normally, it is observed overexpression of cyclin D1 in a variety of human carcinomas 24, 42 is correlated with aggressiveness of the tumor and chemoresistance in ovarian, pancreatic, and nonsmall‐cell lung carcinoma.^43^, ^44^ However, some studies show the reduction in cyclin D1 may be an event interrelated to tumor progression^45^ and the cisplatin resistance.^46^ In our findings, weak cyclin D1 staining was associated with high‐grade serous carcinoma, lymphatic vascular invasion, bilateral involvement, necrosis, and patients’ relapse (Table 2 and Figure 2). Shi and colleagues also observed lower expression of cyclin D1 in serous carcinoma compared with mucinous and clear cell carcinoma subtypes. In addition, functional studies observed that the silencing of the cyclin D1 gene increased migration in breast cancer cells, and cancers with low expression presented were highly infiltrative and presented low recurrence‐free survival, suggesting that cyclin D1 has other functions distinct from the proliferation process.^45^

**Figure 4:**
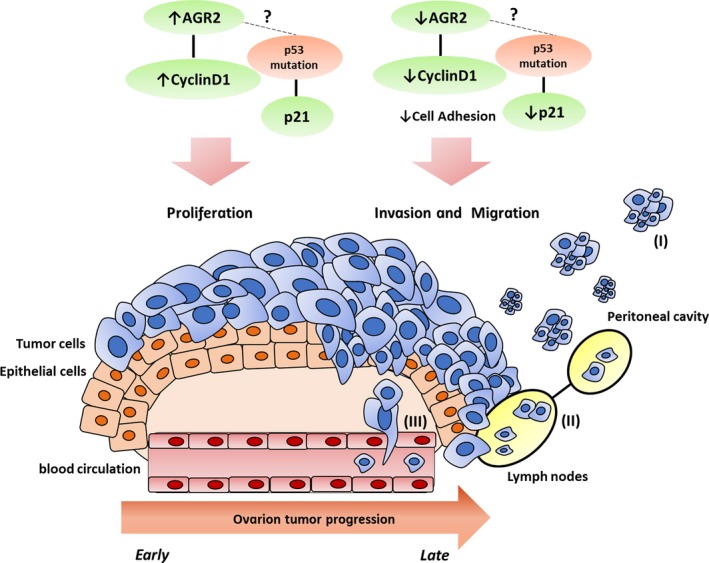
Hypothetic model of AGR2 and cyclin D1 in the progression of ovarian cancer. In early stage of ovarian cancer, increase in AGR2 expression could activate mechanisms of proliferation, such as increase in cyclin D1. In advanced stage, the reduction in AGR2 could activate mechanisms of invasion and metastasis, by reducing adhesion molecules. The metastatic process occurs preferentially to the peritoneal cavity (I) and lymph nodes (II); however, in late stages, the hematogenous metastasis may also occur (III)

Nevertheless, original studies of AGR2 show that this protein has an important function in the preservation of cellular homeostasis, by promoting the differentiation of secretory epithelial cells.^47^ Downregulation of AGR2 seems to be related to tumor progression of tumors originating in secretory organs.^15^, ^18^, ^19^, ^20^, ^21^ Thus, hypothetically, in the early stage of the tumor, increased AGR2 expression could activate proliferative mechanisms, such as increased cyclin D1, but with tumor progression, the reduction in AGR2 could activate mechanisms of invasion and metastasis by reducing adhesion molecules.^21^, ^31^ Loss of AGR2 and cyclin D1 expression during ovarian tumor progression suggests that signaling pathways are altered during the early and advanced stages of the disease and that disbalance in the expression of AGR2 may contribute to the tumor phenotype (Figure 4). To test this hypothesis, functional studies are needed to elucidate the role of AGR2 in carcinogenesis and tumor progression.

Besides this mechanism, it was shown that the expression of AGR2 is positively related to the expression of epithelial markers, and that the reduction in AGR2 induced by TGF‐β created features of mesenchymal cells such as the loss of E‐cadherin, and induction of N‐cadherin. This study showed the importance of AGR2 in maintaining of epithelial phenotypes and suggested that the role of AGR2 in epithelial‐mesenchymal transition is dependent on its localization.^48^ The secreted AGR2 was associated with the extracellular and can influence the activities of multiple extracellular signaling of VEGF and FGF2.^49^

To support our data, we integrate the data obtained by analyzing the protein expression of AGR2 and the other cell cycle protein analysed (p53, p21, and cyclin D1) in public available database. In this approach, we confirmed the lower expression of AGR2 protein as a marker of poor prognosis in epithelial ovarian carcinoma (shorter overall survival). Conversely, p53 mutation and p21 and cyclin D1 protein expression lacked an association with the overall and disease‐free survival. This observation can be probably due to differences in the methodology adopted by our study (IHQ) and TCGA/CPTAC (RPPA and mass spectrometry) and disparities between the cohorts.

Our study observed that the downregulation profile of AGR2, p21, and cyclin D1 and the presence of the suggestive p53 mutation were associated with tumor progression and relapse (Table 2 and Figure 2). Currently, the main factors affecting the prognosis and high mortality of patients with ovarian cancer are advanced stage at diagnosis and primary or secondary chemotherapy drug resistance.^48^, ^49^, ^50^ Thus, the development of more accurate and earlier detection tests for this disease is undoubtedly the number one priority for achieving long‐term reduction in mortality from ovarian cancer, and our results show that the protein evaluation of AGR2, p21, and cyclin D and the suggestive mutation in p53 can differentiate advanced staging and tumor recurrence in response to treatment with carboplatin.

In conclusion, our study observed that the downregulation of AGR2, p21, and cyclin D1 and the presence of the suggestive p53 mutation were associated with tumor aggressiveness characteristics such as progression, invasion, death, relapse, and worse DFS. Interestingly, the expression profiles of the proteins presented herein could predict tumor recurrence after treatment with carboplatin. Currently, relapse in ovarian cancer presents one of the most important limitations in the survival gains of patients, so the evaluation of AGR2 and the proteins of the cell cycle can assistance in the identification of these patients.

## CONFLICT OF INTEREST

The authors declare no conflict of interest.
