# Supplementary figures and images for: Downregulation of AGR2, p21, and cyclin D and alterations in p53 function were associated with tumor progression and chemotherapy resistance in epithelial ovarian carcinoma
Source: Cancer Med. 2018 May 29;7(7):3188–99. doi: 10.1002/cam4.1530 (PMC6051166; doi:10.1002/cam4.1530)

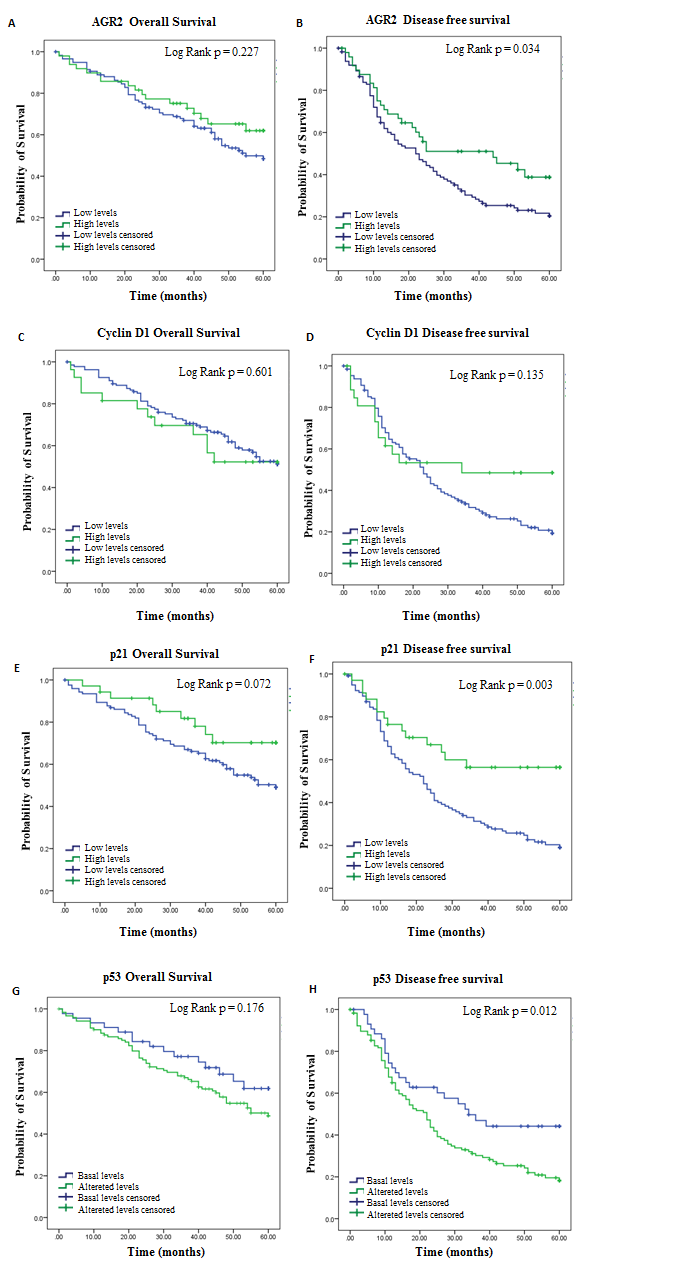

Supplement: Supplementary file 1 [file CAM4-7-3188-s001.tif]
