# Supplementary material for: Downregulation of AGR2, p21, and cyclin D and alterations in p53 function were associated with tumor progression and chemotherapy resistance in epithelial ovarian carcinoma
Source: Cancer Med. 2018 May 29;7(7):3188–99. doi: 10.1002/cam4.1530 (PMC6051166; doi:10.1002/cam4.1530)
Supplement: Supplementary file 2 [file CAM4-7-3188-s002.docx]

Supplementary file 1

**USE OF PUBLIC AVAILABLE DATABASES**

The Proteome data from RPPA (reverse phase protein array) for *TP53* (p53), *CDKN1A* (p21) *and CCND1* (cyclin D1), obtained from TCGA ovarian carcinoma patients, downloaded from cBioPortal for Cancer Genomics website (access 01/09/2018): http://www.cbioportal.org/index.do (option: Thyroid Cancer TCGA, Provisional; Protein expression (RPPA)); Mass spectrometry data from Ovarian Serous Cystadenocarcinoma cancer downloaded in 01/10/2018 from Clinical Proteomic Tumor Analysis Consortium (CPTAC) data portal (https://cptac-data-portal.georgetown.edu/cptacPublic/); TCGA Cancer Proteome Study of Ovarian Tissue: <https://cptac-data-portal.georgetown.edu/cptac/s/S020> (available only for *AGR2 and TP53)* and Exome sequencing data of *TP53* (somatic gene-level non-silent mutation) from TCGA ovarian carcinoma patients, downloaded from cBioPortal for Cancer Genomics website (access 01/10/2018): <http://www.cbioportal.org/index.do>

For inclusion criteria, we used patients diagnosed with primary ovarian carcinoma and follow-up data available (minimum of 6 months and maximum 5 years). Sample size for RPPA: n:354; Mass Spectrometry: n:143; Exome: n:213.

**ANALYSIS PARAMETERS OF PUBLIC AVAILABLE DATA**

Statistical analysis was performed on SPSS (v. 21.0; SPSS, Chicago, IL, USA) software. Gene expression values were dichotomized in bellow and above the first (p53, cyclin D1) or third (AGR2 and p21) quartile, according to the frequency of staining positivity in the internal data IHC. Disease-free and overall survival was estimated and plotted using the Kaplan–Meier method and compared by the log-rank test. A two-tailed P<0.05 value was adopted as significant.

**RESULTS**

**PROTEIN ANALYSIS OF PUBLIC DATA**

| Variables | **RPPA** | | **Mass Spectrometry** | | **Exome** | |
| --- | --- | --- | --- | --- | --- | --- |
|  | Overall survival | Disease free survival | Overall survival | Disease free survival | Overall survival | Disease free survival |
| AGR2 | _ | _ | **0.0309^b^** | 0.4164 | _ | _ |
| p53 | 0.5089 | **0.0110^a^** | 0.3516 | 0.8618 | 0.4623 | 0.1905 |
| Cyclin D1 | 0.1427 | 0.4526 | _ | _ | _ | _ |
| p21 | 0.7113 | 0.1445 | _ | _ | _ | _ |

^a^ higher expression= higher disease-free survival, ^b^ higher expression=higher overall survival. Statistically significant p ≤ 0.05 calculated by log rank test

**SUPPLEMENTARY FIGURE 1**

AGR2 Kaplan–Meier curves analysis for overall and disease-free survival (A, B) respectively, followed by Cyclin D1 (C, D) p21 (E, F) and p53 (G, H) analysis. All p values calculated by the log-rank test.
